# Improving gait classification in horses by using inertial measurement unit (IMU) generated data and machine learning

**DOI:** 10.1038/s41598-020-73215-9

**Published:** 2020-10-20

**Authors:** F. M. Serra Bragança, S. Broomé, M. Rhodin, S. Björnsdóttir, V. Gunnarsson, J. P. Voskamp, E. Persson-Sjodin, W. Back, G. Lindgren, M. Novoa-Bravo, A. I. Gmel, C. Roepstorff, B. J. van der Zwaag, P. R. Van Weeren, E. Hernlund

**Affiliations:** 1 Department of Clinical Sciences, Faculty of Veterinary Medicine, Utrecht University, 3584CM Utrecht, The Netherlands; 2 Division of Robotics, Perception and Learning, KTH Royal Institute of Technology, Stockholm, Sweden; 3 Department of Anatomy, Physiology and Biochemistry, Swedish University of Agricultural Sciences, Uppsala, Sweden; 4 Agricultural University of Iceland, Hvanneyri, Borgarnes Iceland; 5 Department of Equine Science, Hólar University College, Hólar, Iceland; 6 Department of Surgery and Anaesthesiology of Domestic Animals, Faculty of Veterinary Medicine, Ghent University, 9820 Merelbeke, Belgium; 7 Department of Animal Breeding and Genetics, Swedish University of Agricultural Sciences, 75007 Uppsala, Sweden; 8 Livestock Genetics, Department of Biosystems, KU Leuven, 3001 Leuven, Belgium; 9 Genética Animal de Colombia Ltda, Bogotá, Colombia; 10 Equine Department, Vetsuisse Faculty, University of Zurich, Winterthurerstrasse 260, 8057 Zurich, Switzerland; 11 Inertia Technology B.V., Enschede, The Netherlands; 12 Agroscope – Swiss National Stud Farm, Les Longs-Prés, 1580 Avenches, Switzerland; 13 Institute of Genetics, Vetsuisse Faculty, University of Bern, Bremgartenstrasse 109a, 3012 Bern, Switzerland

**Keywords:** Animal physiology, Biomechanics

## Abstract

For centuries humans have been fascinated by the natural beauty of horses in motion and their different gaits. Gait classification (GC) is commonly performed through visual assessment and reliable, automated methods for real-time objective GC in horses are warranted. In this study, we used a full body network of wireless, high sampling-rate sensors combined with machine learning to fully automatically classify gait. Using data from 120 horses of four different domestic breeds, equipped with seven motion sensors, we included 7576 strides from eight different gaits. GC was trained using several machine-learning approaches, both from feature-extracted data and from raw sensor data. Our best GC model achieved 97% accuracy. Our technique facilitated accurate, GC that enables in-depth biomechanical studies and allows for highly accurate phenotyping of gait for genetic research and breeding. Our approach lends itself for potential use in other quadrupedal species without the need for developing gait/animal specific algorithms.

## Introduction

The horse, *Equus ferus caballus,* is a remarkable animal athlete with unique anatomical and physiological features that allow highly efficient locomotion realized by a variety of gaits. The different gaits are characterized by specific limb movement sequences, which can be described by spatiotemporal biomechanical parameters^1^. These gait patterns are orchestrated by the nervous system. Networks of interspinal neurons, known as central pattern generators (CPGs), produce rhythmic output that coordinates the limbs and provides punctual control of hundreds of skeletal muscles^2^. Walk, trot and canter are the standard gaits of all horses, but some breeds can display additional gaits. A gene mutation (DMRT3_Ser301STOP) that alters the CPGs has been found in some breeds such as the Icelandic horse, permitting exhibition of additional gaits, like the tölt and pace^3^. These so-called ‘gaited’ breeds have been purposefully bred, most likely for the extra comfort these gaits offer to the rider^4^.

Scientific work on gaits in animals was pioneered by Milton Hildebrand^5^. In a ground-breaking article published in Science in 1965 he described a gait classification paradigm for quadrupeds based on two kinematic gait parameters—relative hind limb stance duration (duty factor) and lateral advanced placement^5^. Hildebrand and others, using manually and subjectively digitized high-speed films, have categorized quadrupedal locomotion into walking and running, and into symmetrical and asymmetrical gaits. These relatively simple classification categories have, however, been questioned as to how accurate they are in reliably distinguishing gaits and to what extent they can explain the complex gait patterns generated by the multiple components of the locomotor apparatus of quadrupedal animals. More recently, multidimensional approaches have been used^6,7^, challenging the old dogma.

The introduction of sensor technology in motion studies allows easy collection of large amounts of high-resolution, high-sample rate data^8^ that can be used to train models for gait classification. Here, we used sensor-based data to investigate the accuracy of different classification models, based on machine learning technology. We have focused on two main methodologies to train classification models. One approach used a previously described algorithm^9^ for feature extraction by calculating locomotion parameters from limb-mounted IMU sensors. Using this approach, several models were trained, demonstrating that the most important feature for proper gait classification in this approach is the (complex) interlimb relation. Application of this technique largely confirmed Hildebrand’s theory, but also resulted in more accurate gait classification than the original approach, allowing for a refinement of the concept. Further, we have shown that a deep learning approach on raw IMU sensor data (i.e. not based on feature extraction) using a long-short term memory (LSTM) network can also be used to achieve high accuracy in gait classification. This indicates that the time-consuming task of generating animal-specific and gait-specific algorithms can be overcome and opens wide perspectives for the application of this approach in other animal species that are much less researched than the horse.

In this study we aimed at describing a method for accurate and fully automated gait classification in horses using data containing a unique number of gait varieties. We hypothesized that accurate gait classification could only be achieved using higher dimensional models. We further hypothesized that it would be possible to use deep learning techniques not requiring feature extraction, which are hence directly applicable to gait studies in other, less researched species, with similar accuracy.

## Results

**Figure 1:**
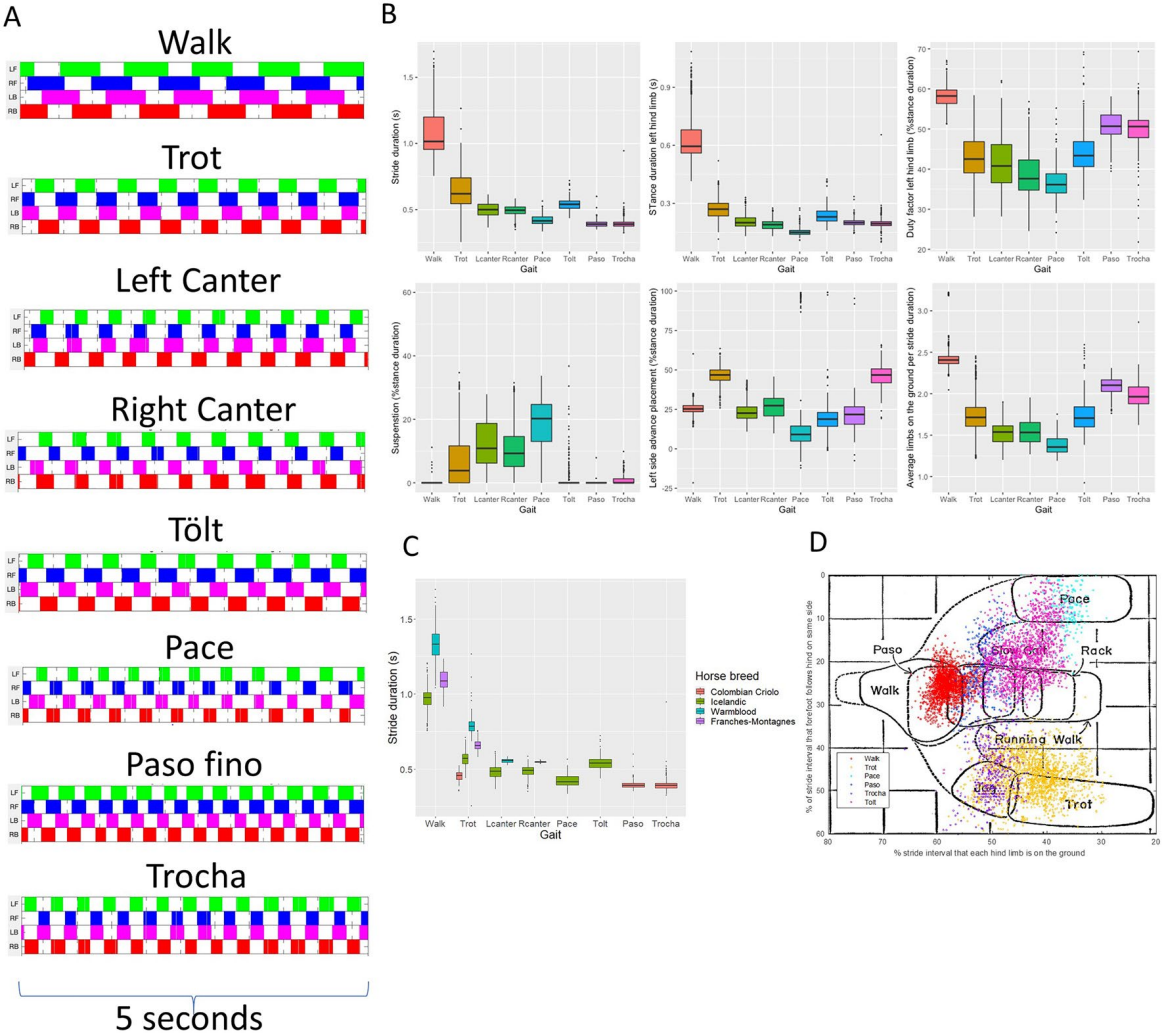
Descriptive results for stride parameters for all gaits. (**A**) Footfall pattern of each different gait. White: swing phase; color: stance phase. *LF* Left front, *RF* Right front, *LB* Left hind, *RB* Right hind. (**B**) Different stride parameters, calculated from the limb-mounted IMUs, grouped by gait. (**C**) Stride duration clustered by gait and horse breed. Note the specific breed characteristics (i.e., clustering). (**D**) Our data overlapping the original Hildebrand 1965 plot where x axis: diagonal advanced placement, y axis: lateral advanced placement.

The footfall pattern and the sequence of footfalls can be defined for each gait (Fig. 1A). Some specific features of the gaits can easily be identified, such as symmetry and laterality. However, for some gaits such as walk, tölt and paso fino, these variables do not fully discriminate between the gait classes. Similarly, other discriminating features, such as the stride temporal variables (Fig. 1B, Table 1), can be differentiating enough for some gaits, such as for example stance duration for the walk (0.65 ± 0.12 s) and the trot (0.28 ± 0.05 s), but in other gaits some of these features overlap, such as stance duration for paso fino (0.21 ± 0.01), and trocha (0.2 ± 0.03). This indicates that multidimensional classification models are required for the comprehensive classification of all gaits.

**Table 1:** Descriptive statistics, mean and standard deviation (SD) of some of the stride temporal variables for each gait.

	Strides	Stride duration (s)		Stride frequency (Hz)		Stance duration (s)^a^		Duty factor^a^	
		Mean	SD	Mean	SD	Mean	SD	Mean	SD
Walk	1966	1.80	0.17	0.95	0.14	0.65	0.12	60.60	1.85
Trot	1932	0.63	0.12	1.64	0.32	0.28	0.05	44.20	4.76
Lcanter	519	0.50	0.05	2.03	0.22	0.19	0.02	39.10	3.58
Rcanter	483	0.49	0.04	2.05	0.18	0.20	0.02	40.00	3.28
Tölt	1572	0.42	0.04	2.39	0.19	0.15	0.02	36.10	2.63
Pace	277	0.54	0.04	1.87	0.13	0.24	0.03	44.20	3.78
Paso	401	0.39	0.03	2.55	0.15	0.21	0.01	53.70	2.49
Trocha	426	0.39	0.04	2.56	0.20	0.20	0.03	51.00	3.34
Total	7576								

^a^Average of all limbs.

Some features are characteristic for specific breeds (Fig. 1C), although some of these differences might also, to some extent, be attributed to conformation and speed. We have made an overlay of our data with data generated by the original classification formula for symmetrical gaits of Hildebrand (Fig. 1D). Although each of our measured gaits falls grossly within the previously described regions, it is evident that the reality is more complex: the overlap is not perfect and the spectra within each gait are broader and less distinct than depicted by the original two-dimensional scheme. Further, the grouping of the different gaits on the 2D plot, such as between pace and tölt, is not clear from the original drawing; these two gaits appear to have a large region of overlap.

### Gait classification based on feature extracted models

**Figure 2:**
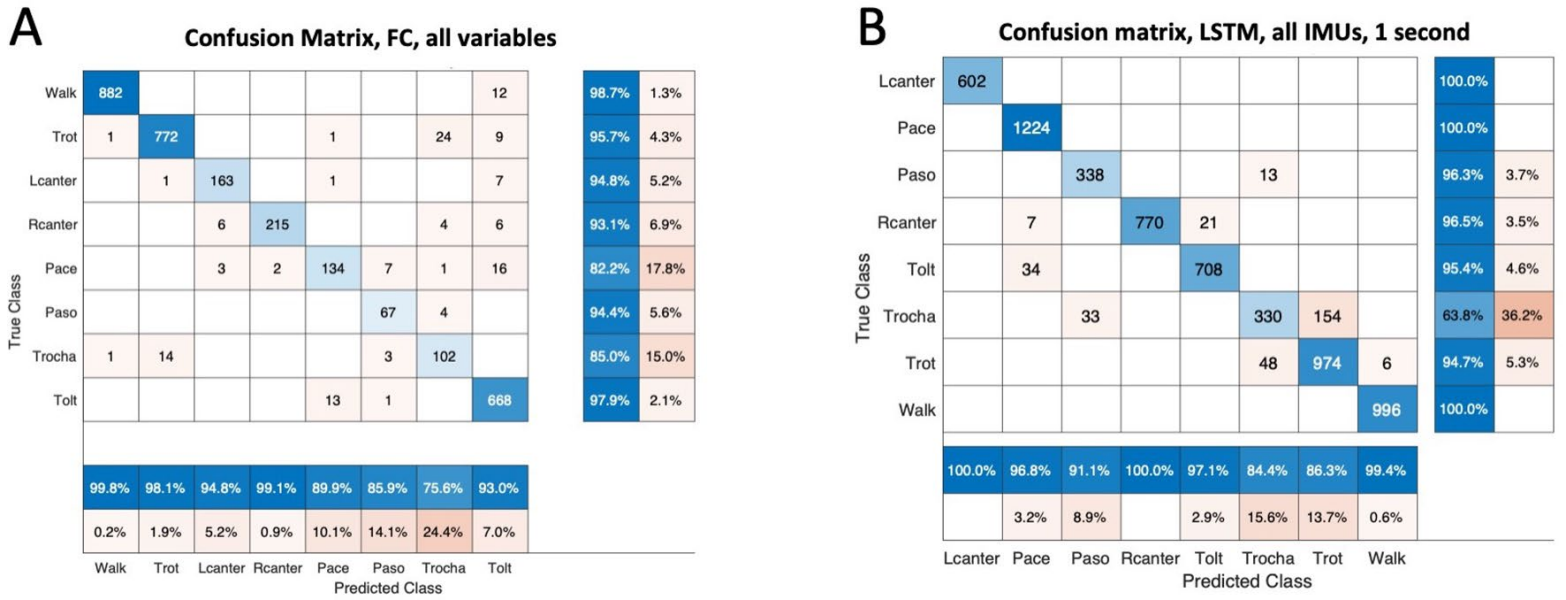
Confusion matrix of the best performing models for two methodologies used; feature extracted models (**A**) and LSTMs based on raw IMU data (**B**). Note the high confusion of the class ‘Trocha’ for both models.

For all the different methods applied, the highest accuracy for classification was obtained when all variables were used, achieving a classification accuracy of 96.7% using a fully connected (FC) artificial neural network followed by 96.3% using the support vector machine model (Table 2). If gait classification was based only on stride variables (e.g., stride duration and duty factor), poor classification accuracy was achieved. With the classification based on the two variables of Hildebrand (duty factor and lateral advanced placement), GC achieved a slightly higher accuracy, peaking at 78.7% using a decision tree. The highest confusion between classes was observed between the gaits trot and trocha for all classification models, followed by the confusion between pace and tölt (Fig. 2). Removing the trocha from the models increased the final accuracy of the best performing FC model to 98.6%.

**Table 2:**
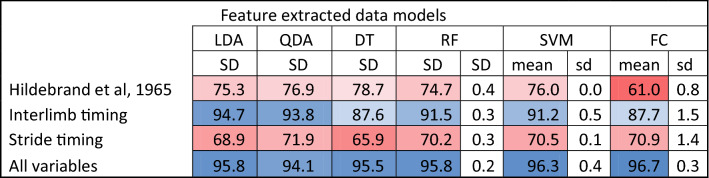
Mean accuracy of five consecutive runs and standard deviation (SD) for feature extracted modes.

*LDA* Linear discriminant analysis, *QDA* Quadratic discriminant analysis, *DT* Decision Tree, *RF* Random Forest, *SVM* Support Vector Machine, *FC* Fully connected ANN.

### Gait classification based on raw IMU data and LSTMs

Classification using LSTMs on the raw normalized sensor data achieved a high classification accuracy, peaking at 95.5% (Table 3). A longer window length had a negative effect, especially when fewer sensors were used (Table 3). Using bidirectional vs unidirectional LSTMs did not affect the general accuracy of each model, although the highest accuracy was achieved with a bidirectional LSTM model.

**Table 3:**
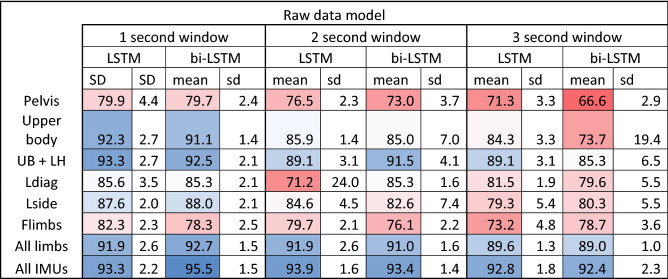
Mean accuracy of five consecutive runs and standard deviation (sd) for LSTM models based on raw IMU data.

Upper body (UB): head, withers and pelvis; *LH* left hind limb, *Ldiag* left diagonal limbs, *Lside* left side limbs, *Flimbs* Front lims.

Gait classification based on a single sensor yielded poor accuracies, peaking at 79.9% only. Training based on sensors mounted on the upper body of horses, mainly head, withers and pelvis, yielded significantly higher accuracies (92.3%) and adding one limb sensor, pushed the accuracies only slightly higher (93.3%), achieving similar accuracies as the models relying solely on all four limbs (92.7%). The highest accuracy was observed using and training the network with the data from all available IMUs, this is, headwithers, pelvis and all four limbs (95.5%). For the best performing models, confusion was highest between the classes trot and trocha, in line with our results from the gait classification models based on feature extraction. Excluding trocha from the data set, yielded a classification accuracy of 98.9%.

## Discussion

In this study we have demonstrated that accurate gait classification in horses can be achieved using state of the art body mounted sensor technology in combination with multiple machine learning data analysis approaches. Through this technology we were able to extend Hildebrand’s original equine gait paradigm from 1965^5^, showing that reality is more complex and ambiguous, and less straightforward than the original concept, as shown in Fig. 1. In fact, this is not unexpected, since Hildebrand’s original model was two-dimensional. Our results confirm that gaits are in fact separated by multidimensional planes and that accurate classification can be achieved for this unique diverse gait data using automated approaches that include minimal preprocessing of the signal.

The human eye has thus far served as the ‘gold standard’ for gait classification. It is clear from the current study, however, that human visual and subjective assessment is not optimal for this purpose. This observation is in line with other studies evaluating human assessment of equine locomotion, mainly in relation to the evaluation of lameness in clinical situations. There too, human subjective assessment proved suboptimal, as it was affected by both the temporal limitations of the human eye^10^ and the proneness to bias^11^.

Our models used in this study open a new world of possibilities, for example for research into genetics of gait. Most equine genetic studies focusing on locomotion, either related to gait^3,12^ or sports performance^13^, require precise phenotyping in order to discriminate between trends in populations or sub-populations. Gait phenotyping is still performed subjectively in most of these studies and thus much less accurate than desirable; we therefore believe that our more accurate methods will allow forthcoming studies to understand the genotype–phenotype association of gaits in greater detail.

Our models using raw sensor data (i.e., LSTMs) achieved a slightly lower accuracy when compared to the feature-extracted models. Nevertheless, the difference was marginal and there is great advantage in using models based on raw sensor data. It is extremely challenging and time consuming to develop specific algorithms for feature extraction^14–16^. These algorithms require validation, and they risk being gait, surface and ultimately, breed specific. Pre-selecting variables also brings the risk of missing information in the data that can be useful for complex classification tasks. When using raw sensor data, the models can be applied to any gait, horse breed and surface, provided that enough labeled training and validation data exist for the development of such models. Hence, this approach is far more widely applicable and opens new possibilities for the study of all gait spectra, not only in the horse, but also in other quadrupedal species.

Window length has a significant effect on the accuracy of our models, and we see a decrease of accuracy with increasing window length (Table 3). We hypothesize that this is related to the fact that segmenting the data into shorter windows results in a larger number of samples that are used as input for model training. Also, longer windows might include more data points where transitions of gait or incorrect strides (e.g. stumbling) occur, and this will ultimately influence the overall accuracy of correctly classifying the entire segment. In theory, it should be possible for the network to learn from longer windows, but we suspect that this would require a larger number of longer samples. For the longer windows, one input sample could contain multiple strides, due to the cyclical nature of the gait data. It is possible that the network learned to disregard the repeated strides of one window if these did not immediately give more information about the coarse-grained class. This way, features capturing the more subtle variation within the strides of one gait might have been lost.

Despite the large influence speed has on temporal variables, such as step duration^17^, our models were able to achieve a high accuracy without a strict control of speed. Hence, we hypothesize that speed might not be a crucial parameter for gait classification. It is therefore questionable if the speed range for each gait in this study did cover the actual variability within each breed. Retraining our models with more data at different speed ranges will improve this in the future.

We have found a high degree of confusion between trot and trocha in our study. This may of course be caused by mislabeling of some of the horses used in the training and validation groups. A recent study described the trocha as often being less ‘clean’ in terms of foot fall timing, possibly related to genetic profiles^12^. Another important issue is the close relation between these two gaits (Fig. 1D). Inclusion of more variables in our models might have allowed for a better separation from the trot, but a close observation of the distribution of trocha versus trot classifications in Fig. 1D also raises doubt whether what is called trocha is not just part of the spectrum of trot, but with a high stride frequency. This warrants further research.

One of the main limitations of the current study is the narrow band of horse breeds used. However, the breeds included in this study were selected to exhibit a variable spectrum of different gaits, and in fact increasing the variation in our population. Overcoming this limitation will be a matter of time, however, because the methods described in this study are adaptive; collection of more data—in other breeds, or even in different species—will lead to better trained models and improved generalization. Future exploration of the machine learning models’ decision process could lead to invaluable insights in locomotor steering.

## Methods

### Data set

**Figure 3:**
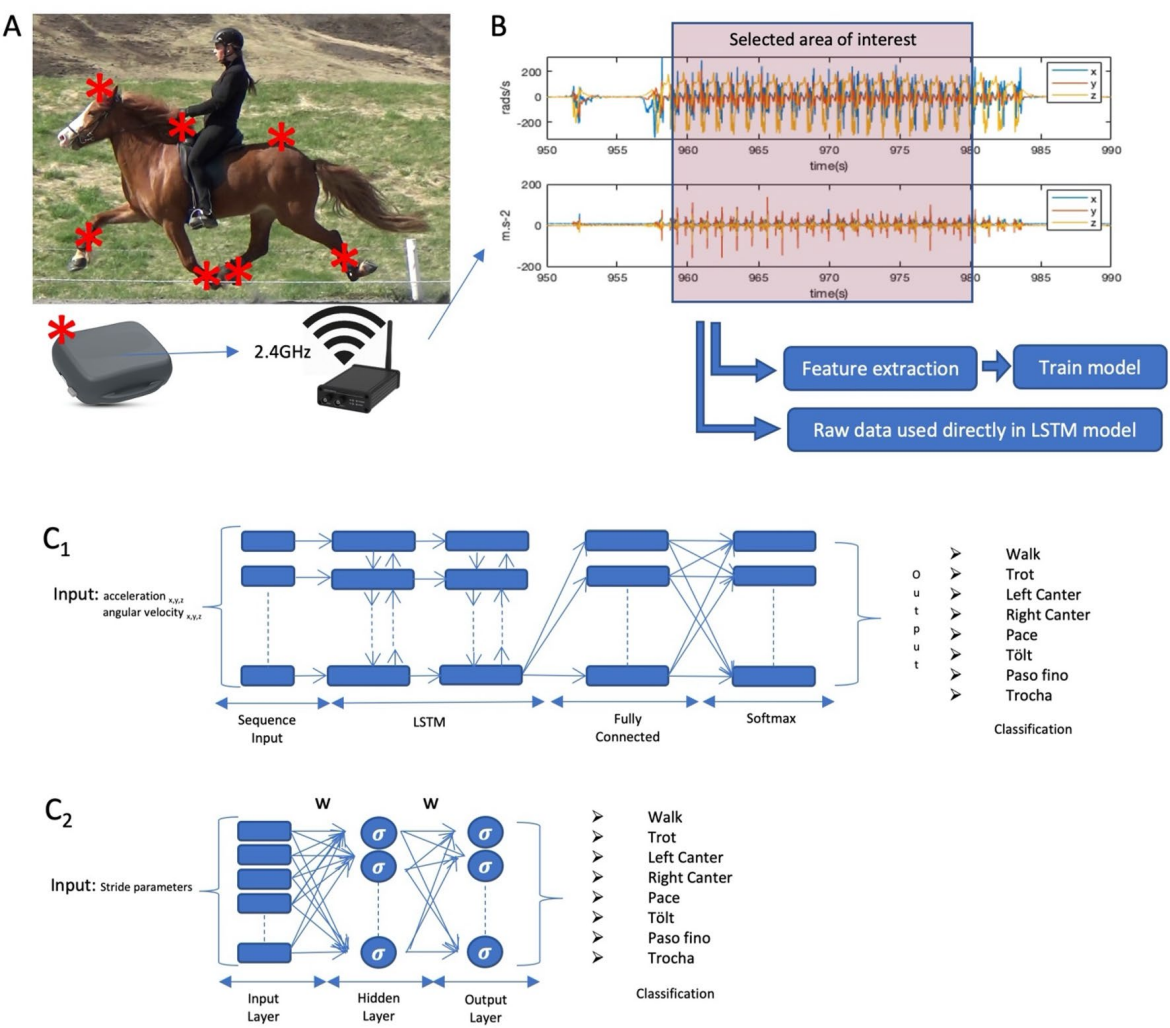
Data collection and analysis procedures. (**A**) One of the study subjects in pace, indicating the location of each IMU sensor in red (*). Raw sensor data was transmitted in real-time from the IMU sensor to a gateway via radio. (**B**) Example of raw data for a segment of IMU data. B_2:_ (**C**) The different ANN training models used; 1: LSTMs, 2: One layer Fully connected.

Data were collected between 2016 and 2019 using seven IMU sensors (Promove-mini, Inertia Technology, The Netherlands) (Fig. 3A). Sensors were attached to the poll, withers and pelvis of all horses, and set to a sampling frequency between 200 and 500 Hz, low-acceleration range of ± 8 g, high acceleration range of ± 100 g and angular velocity of 2000 deg/s. Each limb was also equipped with an IMU sensor, attached to the lateral aspect of the metacarpal/metatarsal bone, and set to a sampling frequency between 200 and 500 Hz, low-acceleration range of ± 16 g, high-acceleration range of ± 200 g and angular velocity of 2000 deg/s. Synchronization between sensors, initial data processing and limb stride parameter calculation were performed as previously described^9, 18^.

Data sets (Table 4) were collected for different research purposes, such as studying objective motion analysis methodology in sound speed-dependent motion patterns in warmblood riding horses and Franches-Montagnes horses and studying gaits and phenotype–genotype associations in gaited horse breeds (Icelandic horses and Colombian horse breeds).

**Table 4:** Description of the data sets.

Breed	Number of trials	Condition	Gaits	Reference
Warmblood	52	In-hand overground and treadmill	Walk, trot, canter	^18–20^
Icelandic horse	44	Ridden and in-hand overground	Walk, trot, pace tölt, canter	^21^
Franches-Montagnes	24	Treadmill	Walk, trot	^22^
Colombian criollo	29	Ridden	Paso fino, trot, trocha	Unpublished data from authors
Total	149			

Includes breed, gaits, number of horses and references to the studies of which the data were used.

For each data set (Table 4), the local Ethics Committee (The Icelandic Food and Veterinary Authority MAST; Ethics Committee for Animal Experiments in Uppsala; Animal Health and Welfare Commission of the canton of Vaud and the ethical committee of Utrecht University in the Netherlands IvD) approved the experimental protocol. All the methods in each individual study where carried out in accordance with the approved guidelines and regulations. Informed consent was obtained from the owner of the animals when needed and no human participants were included in this study. Informed consent for publication has been obtained from the rider in Fig. 3A. All data included in the training and validation of our study were from horses whose athletic performance was normal and they were, to the owners’/trainers’ best knowledge not lame.

### Labeling of the data

For the data sets of the Icelandic horses each measurement was synchronized with a video camera, since each measurement contains several segments of different gaits (walk, trot, pace, tölt and canter). This video was evaluated by a domain expert of gaits of the Icelandic horse (VG), who selected the segments of data that should be used for training and validation. For the Colombian criollo horses, the segments of data used for the analysis were selected based on visual inspection of the footfall pattern during live observation of the trials by an expert in locomotion of this horse breed (MN). For the remaining trials, selection of the segment of each gait was performed by live observation by an expert in equine biomechanics (FSB).

### Preparation of the dataset

Based on the labeled segments of data used for training, a data set was generated. The data set consists of two main parts Fig. 3B, (1) features extracted from the raw IMU data, consisting of stride parameters (Table 5) calculated based on a previously described algorithm^9^ resulting in 7576 strides; (2) segments of the raw IMU data, prepared for the analysis using the LSTMs. Each segment was further cropped into subsections of one, two or three seconds of IMU data. All data were resampled to 200 Hz to match the temporal resolution among all used data sets. A total of 5344 s of raw IMU data were used.

**Table 5:** Description of the features extracted from the IMU sensor data.

	Variable	Unit	Description
Stride timing	Stride duration	s	Duration of one complete stride cycle
	Stance duration		Period of ground contact (weightbearing) of an individual limb
	Stride frequency	Hz	Number of repetitions of the stride unit per second
	Duty factor (relative stance duration)		Duration of stance phase as a proportion of the total limb cycle duration
Interlimb timing	Diagonal advance placement	% of stride duration	Temporal dissociation at hoof contact between diagonal limb pairs
	Lateral advance placement		Temporal dissociation at hoof contact between ipsilateral limb pairs
	Minimum number of limbs on the ground		Minimum number of limbs on the ground per stride
	Maximum number of limbs on the ground		Maximum number of limbs on the ground per stride
	Median number of limbs on the ground		Median number of limbs on the ground per stride
	Quardupedal stance	% of stride duration	Time of simultaneous stance of four limbs
	Tripedal stance		Time of simultaneous stance of tree limbs
	Bipedal stance		Time of simultaneous stance of two limbs
	Single limb stance		Time of simultaneous stance of one limb
	Suspension		Airborne phase of stride where all four limbs are in swing phase and free from weightbearing
	Limb pair overlap LF-RF		Period of synchronous ground contact between LF and RF limbs
	Limb pair overlap LH-RH		Period of synchronous ground contact between LH and RH limbs
	Limb pair overlap LF-LH		Period of synchronous ground contact between LF and LH limbs
	Limb pair overlap RF-RH		Period of synchronous ground contact between RF and RH limbs
	Limb pair overlap LF-RH		Period of synchronous ground contact between LF and RH limbs
	Limb pair overlap RF-LH		Period of synchronous ground contact between RF and LH limbs

*LF* Left front limb, *RF* Right front limb, *LH* Left hind limb, *RH* Right hind limb.

### Data analysis

Data processing, analysis and model training was performed in Matlab 2018b (MathWorks, Natick, Massachusetts, USA). Seven supervised machine learning methods were applied to the gait classification task: linear and quadratic discriminant analysis (LDA and QDA), decision trees, random forest, support vector machine (SVM) a one-layer fully connected (FC) neural network (Fig. 3C2) and a Long-Short Term Memory (LSTM) neural network (Fig. 3C1). With an SVM, as well as with LDA and QDA, we try to learn the decision boundaries that will maximally separate the different classes of our classification problem. In LDA and QDA, we model the data as Gaussian distributed. While in LDA models all classes have the same covariance matrix, QDA has a separate covariance matrix for each class and can thus model more complex decision boundaries. Decision trees are a non-parametric method where the model is trained to split the data according to the most distinguishing features for the different classes. A random forest is an ensemble of decision trees. FC and LSTM are artificial neural network methods, highly parametric as such, that are trained to approximate the function mapping between the input data (raw sensor data or features extracted from sensor data) and the gait class.

The FC model was composed of an input layer of extracted features (Table 5), connected to a hidden layer with a size of 40 neurons, connected to an output layer, representing each one of the output gait classes (walk, trot, left canter, right canter, tölt, pace, trocha and paso fino). The LSTM model was built with an input layer consisting of a sequence of 1, 2 or 3 s of IMU data, connected to two LSTM layers with a width of 500, followed by an FC layer, a softmax layer and a classification layer representing each one of the output gait classes (walk, trot, left canter, right canter, tölt, pace, trocha and paso fino).

For the LSTM, the gyroscope and accelerometer data were normalized between 0 and 1, ensuring that the network will learn the specific gait pattern since we have observed gait-specific characteristics in the magnitude of the signals like for example, higher peak accelerations at trot when compared to walk. Also, gait classes with less data were duplicated in the data set to remove any unbalance present in the data prior to training. Training was performed on a single NVIDIA Tesla K80 GPU with 4992 CUDA cores.

The entire data set was randomly divided in two sub-data sets, one used for training, validation and one for testing. We have ensured that strides of the same horses were never used for training and testing simultaneously, with the goal of avoiding overfitting. Each model was cross-validated using 5 folds and the results presented in Tables 2 and 3 are the mean accuracy and standard deviation of the 5 folds. Based on the best mean validation accuracy, one feature extraction model and one raw data model was selected for testing, the results are presented in Fig. 2A and B.
