# Author Correction: Improving gait classification in horses by using inertial measurement unit (IMU) generated data and machine learning

**DOI:** 10.1038/s41598-021-88880-7

**Published:** 2021-04-26

**Authors:** F. M. Serra Bragança, S. Broomé, M. Rhodin, S. Björnsdóttir, V. Gunnarsson, J. P. Voskamp, E. Persson‑Sjodin, W. Back, G. Lindgren, M. Novoa‑Bravo, A. I. Gmel, C. Roepstorff, B. J. van der Zwaag, P. R. Van Weeren, E. Hernlund

**Affiliations:** 1 Department of Clinical Sciences, Faculty of Veterinary Medicine, Utrecht University, 3584CM , Utrecht, The Netherlands; 2 Division of Robotics, Perception and Learning, KTH Royal Institute of Technology, Stockholm, Sweden; 3 Department of Anatomy, Physiology and Biochemistry, Swedish University of Agricultural Sciences, Uppsala, Sweden; 4 Agricultural University of Iceland, Hvanneyri, Borgarnes Iceland; 5 Department of Equine Science, Hólar University College, Hólar, Iceland; 6 Department of Surgery and Anaesthesiology of Domestic Animals, Faculty of Veterinary Medicine, Ghent University, 9820 Merelbeke, Belgium; 7 Department of Animal Breeding and Genetics, Swedish University of Agricultural Sciences, 75007 Uppsala, Sweden; 8 Livestock Genetics, Department of Biosystems, KU Leuven, 3001 Leuven, Belgium; 9 Genética Animal de Colombia Ltda, Bogotá, Colombia; 10 Equine Department, Vetsuisse Faculty, University of Zurich, Winterthurerstrasse 260, 8057 Zurich, Switzerland; 11 Inertia Technology B.V, Enschede, The Netherlands; 12 Agroscope – Swiss National Stud Farm, Les Longs-Prés, 1580 Avenches, Switzerland; 13 Institute of Genetics, Vetsuisse Faculty, University of Bern, Bremgartenstrasse 109a, 3012 Bern, Switzerland

Correction to *Scientific Reports* 10.1038/s41598-020-73215-9, published online 20 October 2020

The original version of this Article contained errors.

A.I. Gmel was omitted from the author list.

The Author Contributions section now reads:

F.M.S.B., E.H., P.R.W., M.R., conceived the concept. F.M.S.B., E.H., M.R., S.Bj., V.G., M.N., J.V., E.M.P., C.R., A.I.G., carried out the data collection. F.M.S.B. and S.Br. conducted the data analysis. F.M.S.B., E.H., S.Br., G.L., B.J.v.d.Z, P.R.W. W.B. co-wrote the paper. All authors discussed the results and commented on the manuscript.

Additionally, there was a repeated error in the naming of the horse breed “Franches-Montagnes” which was incorrectly given as “Franche Montagne.”

As a result, in Figure 1C, the key describing “Franches Montagne” now reads “Franches-Montagnes.”

The original Figure 1 and accompanying legend appear below.

**Figure 1:**
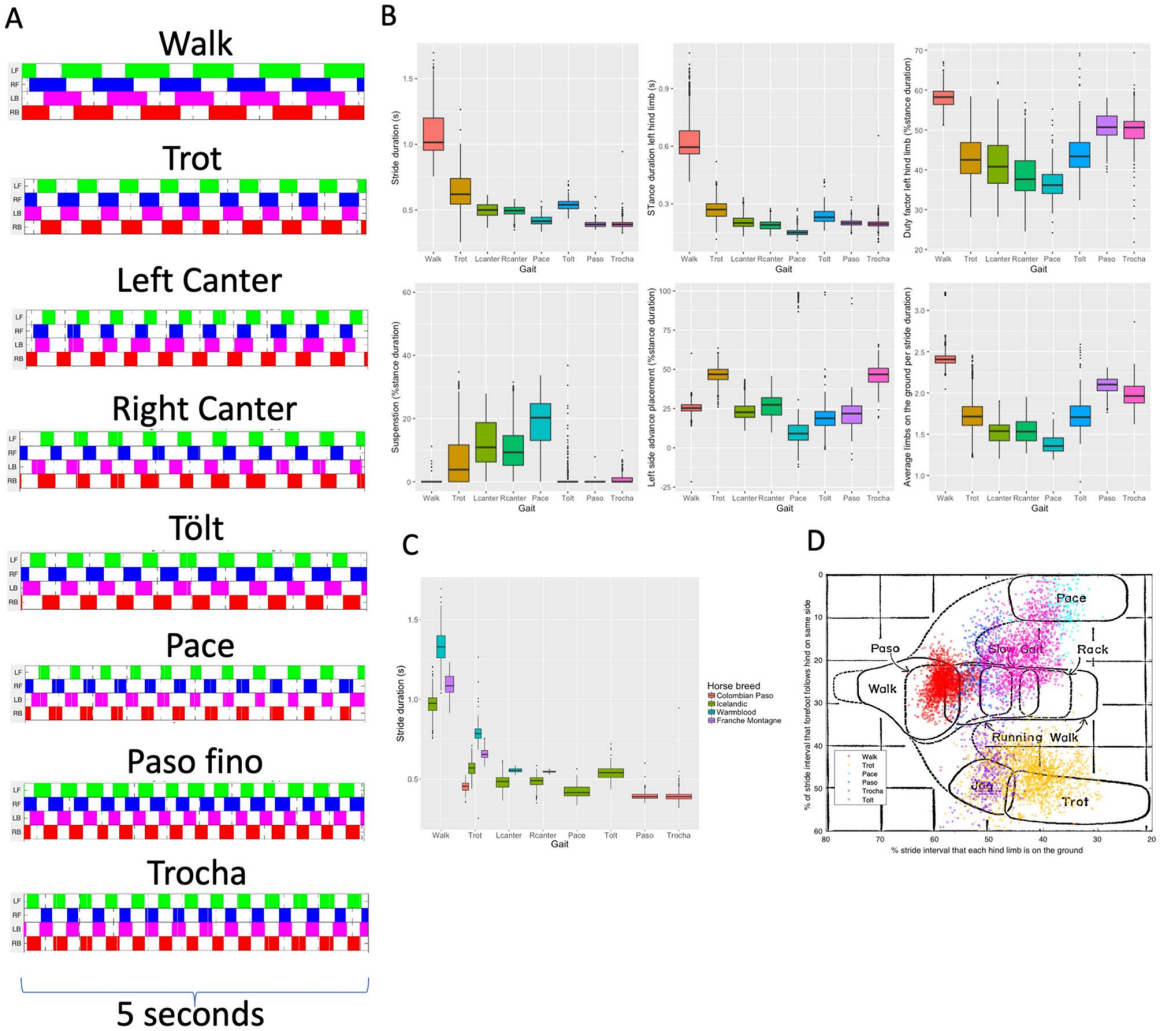
Descriptive results for stride parameters for all gaits. (**A**) Footfall pattern of each different gait. White: swing phase; color: stance phase. *LF* Left front, *RF* Right front, *LB* Left hind, *RB* Right hind. (**B**) Different stride parameters, calculated from the limb-mounted IMUs, grouped by gait. (**C**) Stride duration clustered by gait and horse breed. Note the specific breed characteristics (i.e., clustering). (**D**) Our data overlapping the original Hildebrand 1965 plot where x axis: diagonal advanced placement, y axis: lateral advanced placement.

In the Methods, subheading “Data set”,

“Data sets (Table 4) were collected for different research purposes, such as studying objective motion analysis methodology in sound speed-dependent motion patterns in warmblood riding horses and Franche Montagne horses and studying gaits and phenotype–genotype associations in gaited horse breeds (Icelandic horses and Colombian horse breeds).”

now reads:

“Data sets (Table 4) were collected for different research purposes, such as studying objective motion analysis methodology in sound speed-dependent motion patterns in warmblood riding horses and Franche-Montagnes horses and studying gaits and phenotype–genotype associations in gaited horse breeds (Icelandic horses and Colombian horse breeds).”

Furthermore, in the same section,

“For each data set (Table 4), the local Ethics Committee (The Icelandic Food and Veterinary Authority MAST; Ethics Committee for Animal Experiments in Uppsala; Animal Health and Welfare Commission of the canton of Zurich and the ethical committee of Utrecht University in the Netherlands IvD) approved the experimental protocol.”

now reads:

“For each data set (Table 4), the local Ethics Committee (The Icelandic Food and Veterinary Authority MAST; Ethics Committee for Animal Experiments in Uppsala; Animal Health and Welfare Commission of the canton of Vaud and the ethical committee of Utrecht University in the Netherlands IvD) approved the experimental protocol.”

In Table 4, the “Breed” in column 1,

“Franche montagne”.

now reads:

“Franches-Montagnes”.

Lastly, the Acknowledgements section was incomplete.

“The Pálmi Jónsson's Nature Conservation Fund, the Swedish Norwegian foundation for Equine research (H-17-47-303), the research council of Norway (grant number: HE 284171), and FORMAS (2018-00737 and 2016-00947) funded this study. EquiMoves E! 12304 Eurostars—The Eurostars Programme is powered by EUREKA and the European Community.”

now reads:

“The Pálmi Jónsson's Nature Conservation Fund, the Swedish Norwegian foundation for Equine research (H-17-47-303), the research council of Norway (grant number: HE 284171), and FORMAS (2018-00737 and 2016-00947) funded this study. EquiMoves E! 12304 Eurostars—The Eurostars Programme is powered by EUREKA and the European Community. The Swiss federal Office for Agriculture funded the data collection for the Franches-Montagnes data under contract number 625000469. We thank all persons such as horse handlers, owners and technical staff involved in data collection.”

These errors have now been corrected in the PDF and HTML versions of the Article.

